# BanglaVoice: A curated sentence-level annotated dataset for active, passive, and middle voice in Bangla with baseline classification benchmarks

**DOI:** 10.1016/j.dib.2026.113078

**Published:** 2026-07-15

**Authors:** Md. Jahidul Alam, Zannatul Mawa Koli, Labony Sur, Zahara Al Zarin, Most. Hasna Hena, Tapasy Rabeya

**Affiliations:** a Department of Computer Science and Engineering, Chandpur Science and Technology University, Bangladesh; b Department of Computer Science and Engineering, Daffodil International University, Bangladesh

**Keywords:** Bangla NLP, Grammatical voice annotation, Voice classification, Sentence corpus, Low-resource language dataset, Morpho-syntax

## Abstract

BanglaVoice is a curated sentence-level dataset for grammatical voice analysis in Bangla, comprising 4397 annotated sentences categorized as Active (1459), Passive (1531), and Middle (1407). Each instance consists of a structurally complete clause containing a single dominant finite verb, accompanied by its English translation and a categorical voice label. Sentences were selectively compiled from publicly accessible Bangla digital sources published between 2023 and 2025 and underwent systematic cleaning, de-duplication, orthographic normalization, and UTF-8 standardization. Voice annotations were assigned using linguistically defined criteria and validated through multi-annotator agreement with majority voting. The dataset exhibits balanced class distribution and natural language characteristics, including a Zipfian rank–frequency distribution (s ≈ 0.98–0.99; R² ≈ 0.99) and substantial lexical diversity (3234 unique tokens). Baseline experiments using six supervised classifiers are also provided, with LinearSVC achieving 93.18% accuracy and 93.10% F1-score, establishing reproducible reference benchmarks for future Bangla grammatical voice classification research. BanglaVoice is released as an open-access resource to support morpho-syntactic research and voice-aware modelling in Bangla natural language processing.

Specifications Table

**Table:** 

Subject	Computer Sciences
Specific subject area	Bangla Natural Language Processing (NLP); Grammatical Voice Annotation; Morpho-syntax; Argument Structure Analysis; Low-Resource Language Datasets.
Type of data	Text Files (xlsx-formatted)
Data collection	The dataset was compiled from publicly accessible Bangla digital sources, including news portals, magazines, blogs, and literary platforms (2023–2025). Sentences containing a single finite verb were systematically selected to ensure clear grammatical voice realization (Active, Passive, Middle). Entries were cleaned, de-duplicated, orthographically standardized, and normalized to Unicode-compliant Bangla script (UTF-8). Voice labels were assigned using linguistically defined rule-based criteria and validated by multiple native Bangla speakers through majority voting. Data pre-processing and annotation management were conducted using Python (v3.10) with Pandas and NumPy.
Data source location	Bangla text data collected from publicly accessible digital sources including news portals, magazines, blogs, and literary platforms originating primarily from Bangladesh.
Data accessibility	Repository name: Mendeley Data Data identification number: 10.17632/ts6547j6sc.2 Direct URL to data: https://data.mendeley.com/datasets/ts6547j6sc/2 Instructions for accessing these data: Open access; the dataset is freely available for download from the Mendeley Data repository via the provided DOI link without registration or access restrictions.
Related research article	BanglaVerb: A Sentence-Level Dataset for Transitivity Classification in Bangla NLP [1]. BanglaVerb is methodologically related but contains a separate dataset and a different annotation layer.

## 1 Value of the Data

• BanglaVoice is useful for researchers working on Bangla NLP, grammatical voice annotation, morpho-syntax, corpus linguistics, and low-resource language datasets. It supports analysis of how Bangla encodes agent–patient relations across Active, Passive, and Middle voice constructions.
• The dataset can be used for semantic role labelling (SRL) evaluation. For example, a Bangla SRL system can be tested on whether it correctly identifies the agent in Active sentences such as “” — Rahul reads the book, and in Passive sentences such as “ ” — the book was read by Rahul, where the agent appears in a “”-marked phrase.
• BanglaVoice can be used to evaluate voice sensitivity in Bangla NLG systems. Since the BanglaNLG benchmark [2] includes Bangla machine translation and question answering tasks but does not explicitly control for grammatical voice, BanglaVoice can serve as a diagnostic probe set to test whether models such as BanglaT5 preserve correct agent–patient mappings when sentences shift among Active, Passive, and Middle voice.
• The supervised classification experiments provide quantitative baseline benchmarks. Researchers can directly reuse BanglaVoice by taking the Bangla sentence column as input and the Voice column as the target label for three-class grammatical voice classification. Six baseline models were evaluated in this study, and the best-performing model, LinearSVC, achieved an accuracy of 0.9318 and an F1-score of 0.9310, providing reference scores for future transformer-based, syntax-aware, or cue-controlled Bangla grammatical voice classification models.
• BanglaVoice complements BanglaVerb [1] by contributing a distinct annotation layer. While BanglaVerb focuses on transitivity classification, BanglaVoice focuses on grammatical voice distinctions among Active, Passive, and Middle constructions. The two resources are distinct in scope and content, and BanglaVoice does not reuse sentence instances or annotation labels from BanglaVerb.

## 2 Background

Bangla (Bengali) is among the most widely spoken languages globally, yet it is still treated as comparatively low-resource in Natural Language Processing (NLP) due to the limited availability of large, linguistically structured datasets and robust tools for high-level language understanding [3]. A major consequence of this resource gap is that many core applications such as machine translation, information extraction, question answering, and speech-driven systems, remain less accurate and less inclusive for Bangla-speaking users than for high-resource languages [3].

In recent years, the Bangla NLP community has accelerated dataset creation to reduce this scarcity. Public data articles have introduced task-focused resources covering sentence transformation/classification (BTSD) [4], tense classification (BanglaTense) [5], sarcasm detection from social media (BanglaSarc3) [6], relation extraction (Bangla-REX) [7], and speech emotion recognition (BanglaSER) [8]. Alongside these datasets, transformer-based Bangla models (e.g., Bangla-BERT) have strengthened downstream performance and broadened evaluation possibilities [9]. Collectively, these efforts show steady progress toward data-driven Bangla NLP, but they also highlight that grammar-sensitive phenomena are still underrepresented in openly available, sentence-level resources [3]. A recent data article, BanglaVerb, demonstrated the value of explicitly modeling verb-related structure by providing sentence-level labels for transitive vs. intransitive constructions and enabling verb-aware modeling for Bangla [1]. However, transitivity is only one dimension of verb structure; voice alternations (Active, Passive, Middle) constitute another fundamental mechanism through which languages express argument realization and event interpretation. BanglaVerb was published earlier in 2026 and is cited here as a related but non-overlapping resource. No BanglaVerb instances or transitivity labels were reused in BanglaVoice.

Cross-linguistic research shows that Passive and Middle are not merely stylistic variants of Active, but correspond to distinct morpho-syntactic configurations with systematic effects on argument suppression, agentivity, and clause semantics [[10], [11], [12]]. Passive constructions typically suppress or demote external arguments, while middle constructions are associated with characteristic shifts in agentivity and internal-argument prominence, producing stable differences in interpretation and structure [10,12]. From an NLP perspective, making voice explicit is crucial because many downstream systems depend on accurate recovery of “who did what to whom.” Voice influences subject–object alignment and event structure, affecting machine translation and question answering; it also shapes dependency parsing and morpho-syntactic tagging, where correct identification of verb forms and argument realization is required [3]. Without voice-aware resources, models may conflate agent/patient roles across active vs. non-active clauses, which can propagate errors into information extraction, semantic modelling, and speech interfaces that require grammatically coherent outputs.

Despite the linguistic and computational importance of voice, publicly available Bangla datasets with sentence-level Active–Passive–Middle labels remain extremely limited. This study introduces BanglaVoice, a curated dataset of Bangla sentences annotated for grammatical voice classification. The dataset is intended to support morpho-syntactic analysis and enable the development of voice-aware Bangla NLP models for tasks where argument structure and event interpretation are central.

## 3 Data Description

The BanglaVoice dataset consists of sentence-level Bangla text annotated for three grammatical voice categories: Active, Passive, and Middle. Each row represents a single structurally complete sentence containing one dominant finite verb and its corresponding voice label. The dataset is distributed in spreadsheet format (.xlsx) and contains 4397 unique sentence instances organized in a structured tabular layout. All textual content is encoded in UTF-8 Unicode and normalized using standard Bangla script to ensure cross-platform compatibility and seamless integration with computational pipelines.

The dataset comprises three columns named Sentence, Sentence_en, and Voice, as summarized in Table 1. The *Sentence* column contains the original Bangla sentence annotated for grammatical voice. The *Sentence_en* column provides the English translation to support cross-lingual analysis and interpretability. The *Voice* column assigns each sentence to one of three categorical classes: Active, Passive, or Middle. This structured schema enables direct reuse for supervised classification, morpho-syntactic analysis, and multilingual evaluation tasks.

**Table 1:** Dataset schema, attributes, and possible values.

Column Name	Description	Data Type	Possible Values	Example
Sentence	Original Bangla sentence annotated for grammatical voice. Each sentence contains a clearly identifiable finite verb used to determine voice category.	Text	Bangla sentence (UTF-8 encoded)	
Sentence_en	English translation of the corresponding Bangla sentence, provided to support cross-lingual analysis and interpretability.	Text	English sentence	Rahul is reading a book.
Voice	Grammatical voice classification assigned to the sentence.	Categorical	Active / Passive / Middle	Active

To improve dataset usability and reliability, several quality checks were applied before final release. These included removal of duplicate and incomplete sentence entries, exclusion of structurally ambiguous clauses, Unicode/UTF-8 normalization, manual verification of Bangla orthography, preservation of voice-relevant grammatical markers such as “”, and “”, and validation of labels through multi-annotator agreement. The released spreadsheet files were also checked to ensure that Bangla text, English translations, and categorical voice labels were consistently aligned across rows. Representative examples in Table 2 illustrate how Active, Passive, and Middle instances were identified using explicit annotation cues. The examples illustrate how Active constructions are identified through an explicit agent performing an action, Passive constructions through patient foregrounding and agent-marking cues such as “”, and Middle constructions through event- or process-oriented interpretations where the agent is not explicitly expressed. The released spreadsheet files were verified as Unicode-compliant UTF-8 Bangla text; any visible irregularities in the PDF are rendering or font-display artifacts rather than encoding errors in the underlying dataset.

**Table 2:** Representative annotated instances from the BanglaVoice dataset with voice-specific annotation cues.

**Fig. 1:**
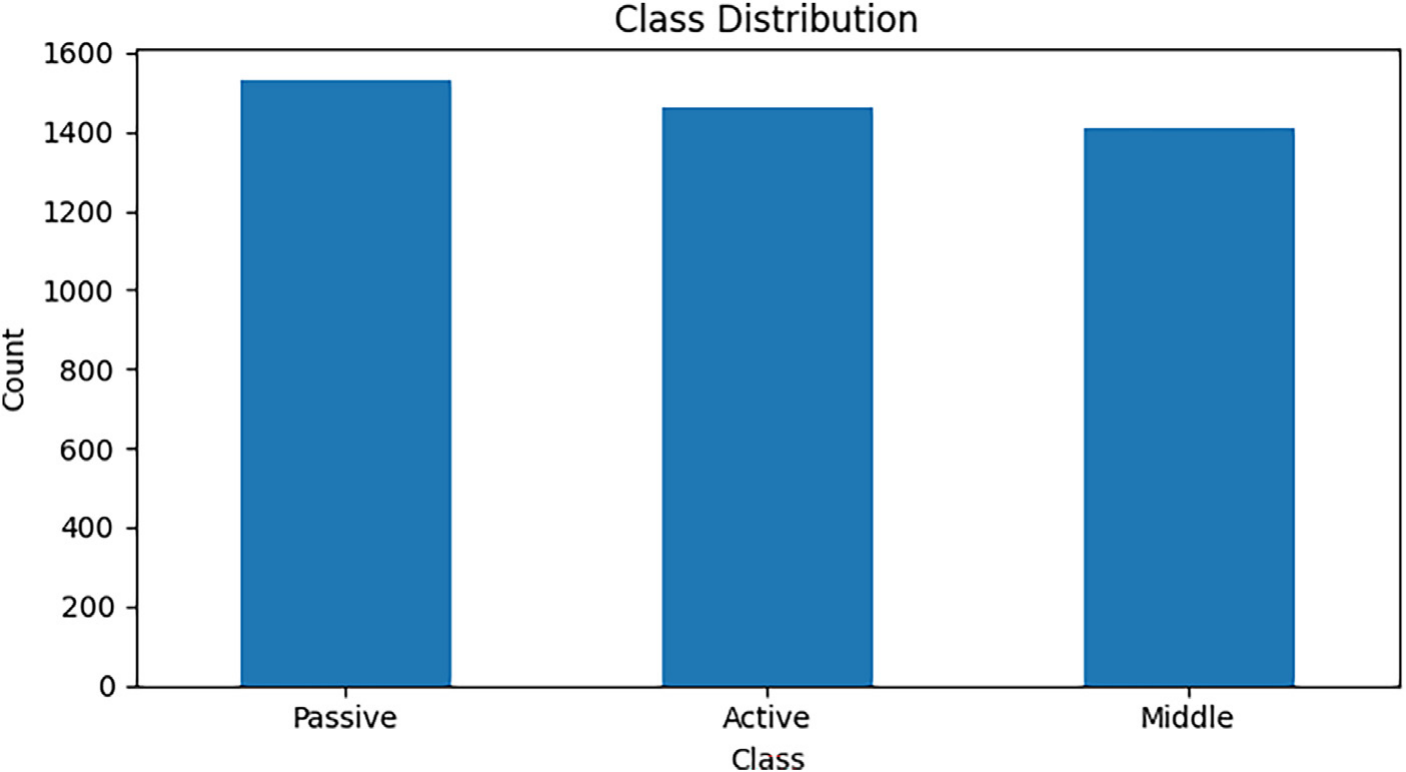
Class distribution of active, passive, and middle sentences in the BanglaVoice dataset.

The distribution of sentence instances across grammatical voice categories is shown in Fig. 1. The dataset contains 1459 Active sentences (33.19%), 1531 Passive sentences (34.82%), and 1407 Middle sentences (31.99%), totalling 4397 unique entries. The three categories exhibit near-balanced representation, with only minor proportional differences across classes. This distribution supports comparative morpho-syntactic analysis and supervised modelling without substantial class imbalance.

**Fig. 2:**
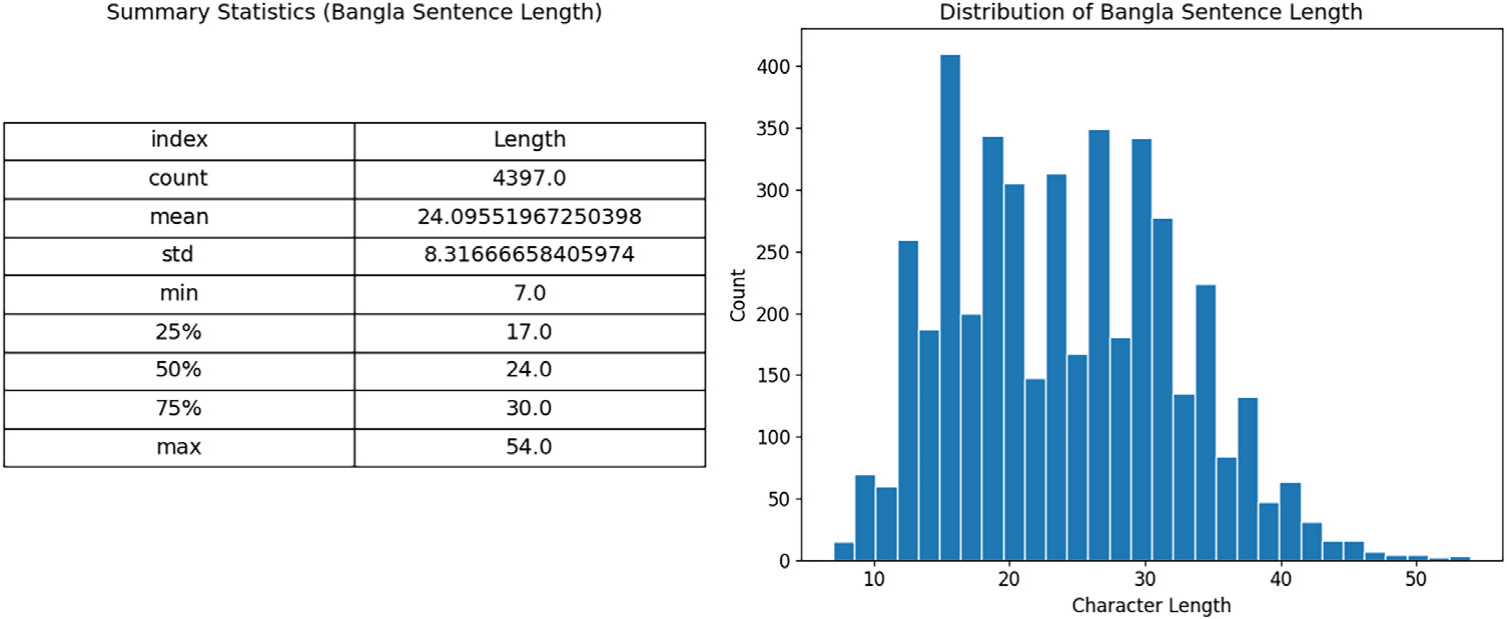
Summary statistics and distribution of Bangla sentence length (character-based) in the BanglaVoice dataset.

Structural characteristics of the dataset were examined using character-level sentence length statistics, as presented in Fig. 2. The dataset contains 4397 sentences, with an average length of 24.10 characters (SD = 8.32). The minimum sentence length is 7 characters, while the maximum reaches 54 characters. Quartile analysis indicates that 25% of sentences contain 17 characters or fewer, the median length is 24 characters, and 75% fall below 30 characters. The histogram in Fig. 2 shows a moderately right-skewed distribution, with the majority of sentences concentrated between approximately 17 and 30 characters. Longer sentences occur less frequently, forming a gradual tail extending toward higher character counts. This distribution reflects structural variability while maintaining overall compact sentence construction across the dataset. In addition to character-based length, token-based sentence length was calculated using a Bangla-compatible delimiter-based tokenizer.

The corpus contains an average of 4.27 tokens per sentence (SD = 1.24), with a median of 4 tokens and a range of 2–10 tokens. Tokenization was performed using a Bangla-compatible delimiter-based tokenizer after Unicode normalization, where tokens were separated mainly by whitespace and punctuation boundaries rather than by subword segmentation. Therefore, the short average token length reflects the dataset design criterion that each selected sentence contains a single dominant finite verb and excludes complex multi-clausal constructions; it is not the result of aggressive token splitting, token merging, or a tokenization artifact. Class-wise token-length averages were 3.84 for Active, 4.89 for Passive, and 4.06 for Middle sentences. Thus, Passive constructions are longer than Active and Middle constructions in both character-level and token-level measurements.

**Fig. 3:**
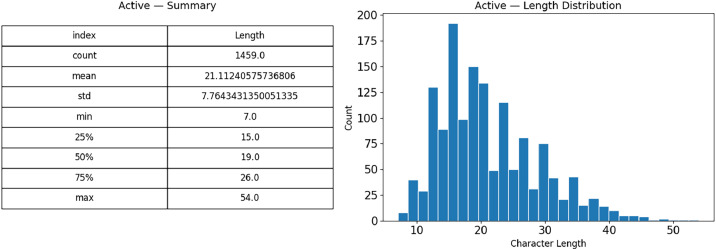
Summary statistics and character-length distribution for active sentences.

**Fig. 4:**
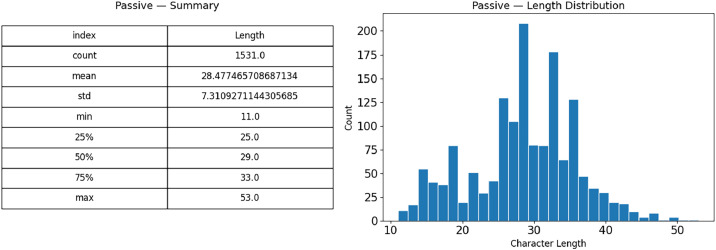
Summary statistics and character-length distribution for passive sentences.

**Fig. 5:**
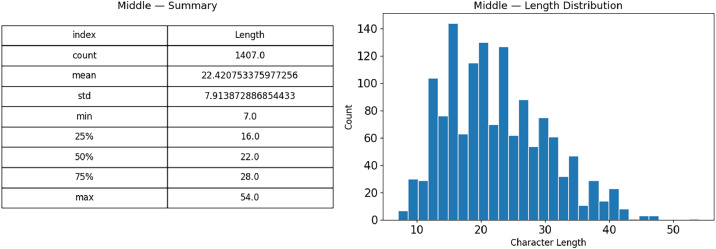
Summary statistics and character-length distribution for middle sentences.

Class-wise structural variation in sentence length is presented in Fig. 3, Fig. 4, Fig. 5. Active sentences (n = 1459) exhibit a mean character length of 21.11 (SD = 7.76) with a median of 19 characters, and an interquartile range from 15 to 26 characters, spanning a total range of 7 to 54 characters. Passive sentences (n = 1531) demonstrate the highest average length, with a mean of 28.48 characters (SD = 7.31) and a median of 29 characters, and an interquartile range from 25 to 33 characters, ranging between 11 and 53 characters. In contrast, Middle constructions (n = 1407) show a slightly higher mean length of 22.42 characters (SD = 7.91) and a median of 22 characters, with quartiles at 16 and 28 characters and the same overall range of 7 to 54 characters. The corresponding histograms indicate that Passive constructions are consistently shifted toward higher character counts, whereas Active and Middle sentences are more concentrated within shorter length intervals, reflecting measurable structural variation across grammatical voice categories.

**Fig. 6:**
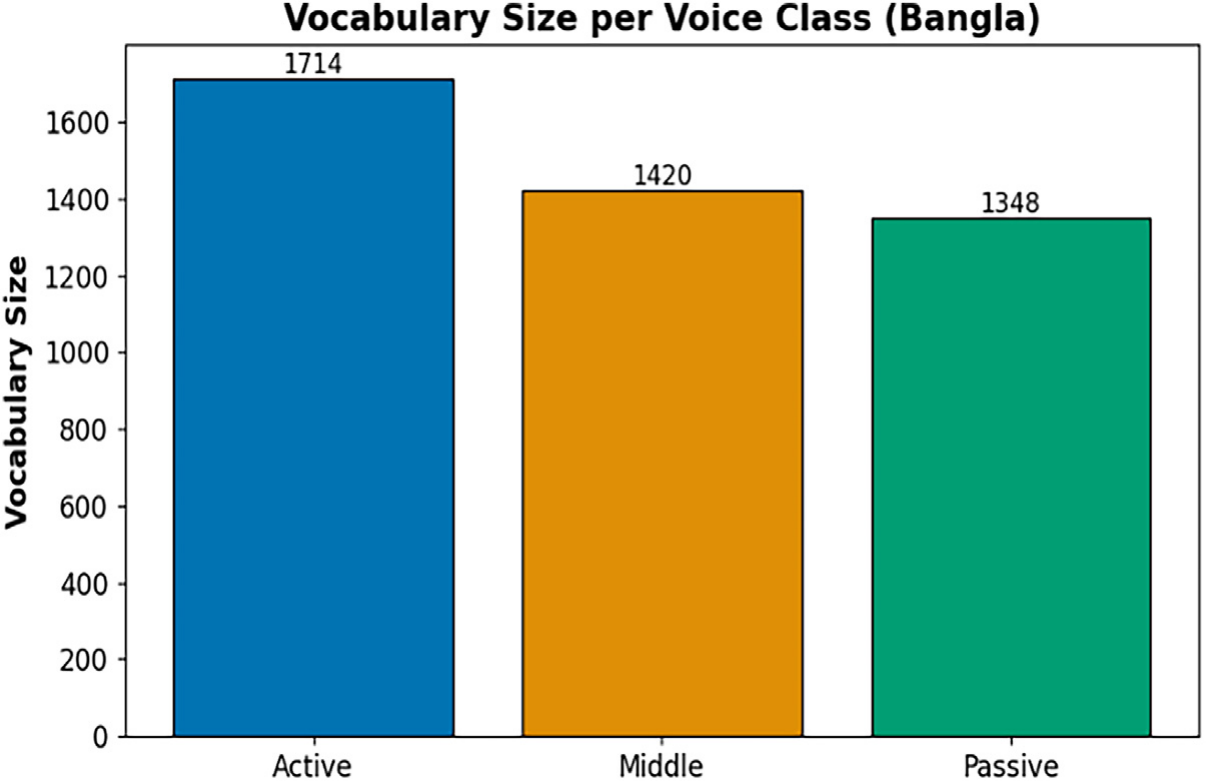
Vocabulary size per voice class in the BanglaVoice dataset.

Vocabulary size across grammatical voice categories is presented in Fig. 6. The Active class contains 1714 unique tokens, followed by Middle with 1420 and Passive with 1348 distinct tokens. In terms of total token frequency, Passive constructions account for the highest number of tokens (7485), whereas Middle and Active contain 5706 and 5603 tokens, respectively. Lexical diversity, measured using the Type–Token Ratio (TTR), differs across categories. The Active class exhibits the highest lexical diversity (TTR = 0.3059), followed by Middle (TTR = 0.2489), while Passive shows comparatively lower diversity (TTR = 0.1801). These results indicate variation in vocabulary distribution and lexical concentration across the three grammatical voice types. The per-class unique token counts are not mutually exclusive because the same token may occur in more than one voice category. Therefore, the class-wise counts in Fig. 6 sum to more than the corpus-level vocabulary size of 3234 unique tokens.

**Fig. 7:**
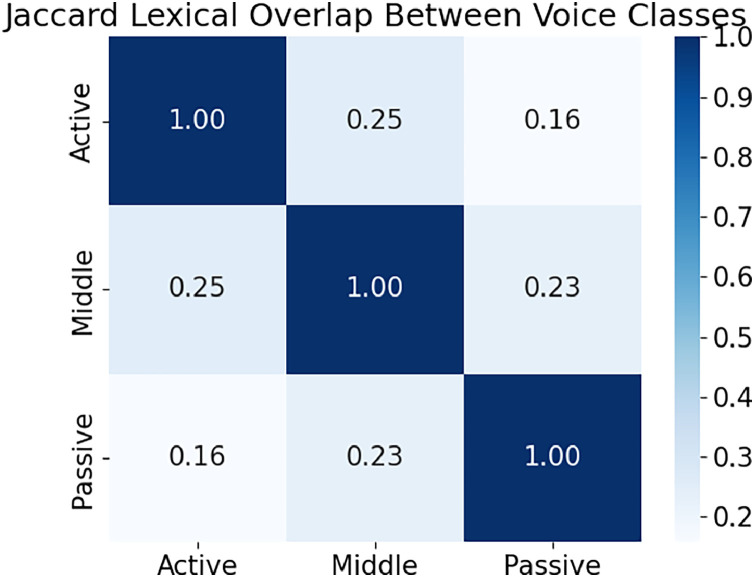
Jaccard similarity matrix showing lexical overlap between active, passive, and middle voice categories.

Lexical overlap between voice categories was further evaluated using Jaccard similarity, as shown in Fig. 7. The highest overlap is observed between Active and Middle constructions (0.2506), followed by Middle and Passive (0.2324). The lowest overlap occurs between Active and Passive categories (0.1581). These values indicate that while the three voice types share a portion of common vocabulary, substantial class-specific lexical variation exists. The comparatively lower similarity between Active and Passive constructions suggests stronger differentiation in lexical usage between these two categories, whereas Middle constructions exhibit moderate overlap with both classes.

Table 3 presents the top 20 most frequent unigrams across Active, Passive, and Middle voice constructions in the Bangla corpus, along with their English functional equivalents. The unigram distribution reveals distinct structural characteristics across the three voice categories.

**Table 3:** Top 20 most frequent unigrams across active, passive, and middle voice constructions in Bangla and their English equivalents.

*Note:* English glosses are functional equivalents used for interpretability; they are not intended as strict one-to-one lexical translations.

In the Active category, high-frequency tokens include pronouns and subject-oriented markers such as  (he),  (I),  (they), and  (you), indicating subject-centered sentence constructions. Content words such as  (song),  (mother), and  (book) also appear prominently, reflecting diverse thematic usage. The Middle voice category is characterized by auxiliary and aspectual markers including  (being),  (done),  (is), and  (been). The frequent occurrence of these tokens suggests a strong presence of progressive and perfect constructions within this category. In contrast, the Passive category is dominated by agent-marking and auxiliary-based grammatical forms, particularly  (by),  (done),  (was), and  (been). The high frequency of  (by), which appears 978 times, highlights the structural prominence of agent-marked passive constructions. Similarly, auxiliary forms such as , and  reinforce the prevalence of formal passive reporting structures. Overall, the unigram distribution demonstrates clear syntactic differentiation among Active, Passive, and Middle constructions. The prominence of auxiliary and agent-marking tokens in the Passive category confirms its structurally repetitive grammatical pattern, while the Active category shows greater lexical diversity through subject and content-word dominance.

Overall, the BanglaVoice dataset provides a systematically curated and linguistically validated resource for sentence-level grammatical voice analysis in Bangla. Through balanced class representation, structural statistics, lexical diversity measurement, overlap quantification, and multi-level n-gram analysis, the dataset demonstrates clear syntactic differentiation across Active, Passive, and Middle constructions. The inclusion of aligned English equivalents further enhances its utility for cross-linguistic and multilingual research. By combining structural transparency with quantified lexical evidence, BanglaVoice establishes a reproducible foundation for computational modelling, morpho-syntactic investigation, and supervised classification tasks in Bangla natural language processing. The dataset is intended to facilitate future research in low-resource language modelling and to support the development of robust, linguistically informed NLP systems.

## 4 Experimental Design, Materials and Methods

**Fig. 8:**
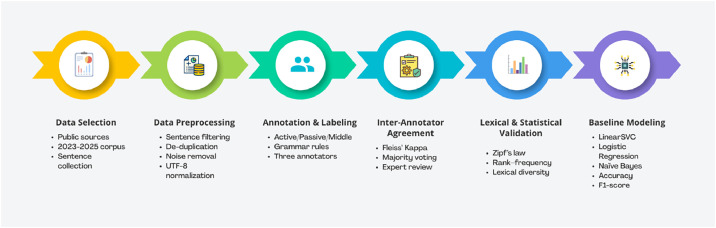
Workflow of the BanglaVoice dataset construction and validation process.

The BanglaVoice dataset was developed through a structured multi-stage framework comprising data selection, pre-processing, manual annotation with inter-annotator agreement analysis, and statistical validation, including lexical evaluation and supervised benchmarking (Fig. 8). The methodological workflow follows a general dataset-construction structure comparable to BanglaVerb [1], including data selection, pre-processing, lexical validation, and supervised baseline benchmarking. However, the present dataset uses newly collected and independently annotated sentence instances, with a distinct annotation scheme focused on grammatical voice rather than transitivity.

### 4.1 Data selection

The BanglaVoice dataset was constructed through a structured data selection process designed to ensure grammatical clarity, structural diversity, and contextual representativeness. Rather than indiscriminate data harvesting, sentences were selectively curated from publicly accessible Bangla digital sources, including news portals, online magazines, blogs, and literary platforms published between 2023 and 2025. Selection criteria focused on identifying structurally complete sentences containing a single dominant finite verb. This constraint was imposed to ensure unambiguous realization of grammatical voice (Active, Passive, or Middle) and to maintain consistency in morpho-syntactic analysis. Sentences containing multiple competing finite verbs, fragmented clauses, or structurally incomplete expressions were excluded to avoid annotation ambiguity. To minimize thematic and contextual bias, sentences were sampled from diverse domains such as current affairs, literature, education, and social commentary. All selected sentences were compiled into a structured spreadsheet format for subsequent pre-processing, normalization, annotation, and statistical validation. This systematic selection strategy ensures that the dataset reflects authentic Bangla usage while maintaining grammatical precision required for voice classification.

### 4.2 Data pre-processing

A structured pre-processing pipeline was applied prior to annotation and statistical analysis to ensure consistency, linguistic validity, and computational reliability throughout the BanglaVoice dataset. The collected sentences were first screened to remove duplicate entries and non-linguistic noise such as incomplete fragments, encoding artifacts, or stray symbols. As shown in Table 4, 4957 candidate sentences were initially collected from four source categories. During pre-processing, 560 sentences were removed because of duplication, incomplete structure, encoding issues, non-linguistic noise, or failure to satisfy the single-dominant-finite-verb criterion. The final dataset therefore contains 4397 sentences, corresponding to an overall attrition rate of 11.30%. Source-wise attrition was monitored to ensure that sentence removal did not disproportionately affect any single source category, thereby reducing the likelihood of systematic source bias during curation.

**Table 4:** Source-wise sentence attrition during pre-processing of the BanglaVoice dataset.

Source	Representative sample URL	Raw collected sentences	Sentences after preprocessing	Removed sentences
News portals	https://www.prothomalo.com/	1165	1025	140
Magazines	https://www.kishoralo.com/	1552	1397	155
Blogs	https://www.sachalayatan.com/	1105	975	130
Literary platforms	https://www.kobitaclub.com/	1135	1000	135
Total		4957	4397	560

As shown in Table 4, magazines formed the largest retained source category, contributing 1397 of 4397 sentences, or 31.77% of the final corpus. However, no source category exceeded one-third-of the dataset, and news portals, blogs, and literary platforms also contributed substantial numbers of sentences. This distribution indicates that the corpus is not uniformly balanced by source type, but all four source categories remain meaningfully represented and support source-level variation in authentic written Bangla usage. Each sentence was then converted into Unicode-compliant Bangla script to resolve potential encoding inconsistencies and ensure cross-platform compatibility. Leading and trailing whitespace was removed, and exact duplicate rows were eliminated to preserve sentence-level uniqueness. Non-essential punctuation marks were stripped where required for lexical analysis, while structurally meaningful markers (e.g., clause boundaries or auxiliary indicators) were retained to preserve grammatical information relevant to voice classification. Orthographic inconsistencies were standardized through rule-based normalization and manual verification, ensuring uniform spelling while maintaining authentic linguistic variation.

Unlike typical text-cleaning pipelines that remove function words, stopwords were deliberately retained during the core pre-processing stage. Since Bangla voice constructions rely heavily on auxiliary verbs and grammatical markers (e.g., “”), preserving these tokens was essential for maintaining syntactic integrity and enabling accurate morpho-syntactic analysis. Following this multi-stage cleaning and normalization process, the dataset was finalized as a structurally consistent and linguistically reliable corpus of 4397 annotated sentences categorized into Active, Passive, and Middle voice constructions. This standardized pre-processing framework provides a robust foundation for statistical evaluation, lexical analysis, and supervised NLP modelling.

### 4.3 Data annotation

All sentences in the BanglaVoice dataset were manually annotated for grammatical voice into three categories: Active, Passive, and Middle. Annotation followed a structured multi-stage protocol to ensure linguistic validity and inter-annotator consistency.

Initially, sentences were screened using rule-based heuristic cues derived from standard Bangla grammar. The annotation guideline instructed annotators to consider verb morphology, auxiliary forms, agent presence or absence, patient/theme foregrounding, “”-marked agents, surface subject role, event/process interpretation, and overall syntactic structure. Structural indicators such as auxiliary forms (), agent markers (), and argument realization patterns were identified to assist annotators. However, these cues were used only as annotation guidance and did not automatically determine the final label; annotators judged the full sentence structure and interpretation before assigning a category.

The guideline defined Active voice as a construction in which the surface subject is the explicit agent or doer of the action, as in “” (“Rahul is reading a book”). Passive voice was defined as a construction in which the patient, theme, or affected entity is foregrounded, while the agent is demoted, omitted, or expressed through an oblique marker such as “”, as in “” (“The book has been read by Rahul”). Middle voice was operationally defined as a construction in which the event or process is foregrounded while the external agent is not explicitly expressed, and the surface subject typically corresponds to the affected entity, theme, or entity undergoing a change/process. For example, “” (“The door opens easily”) was labelled Middle because the sentence describes a process affecting the door without an overt external agent. Sentences were not labelled Middle if an explicit agent performed the action or if a clear Passive construction with agent demotion and markers such as “”, or “” was present.

The three annotators were co-authors of the study and native Bangla speakers from the Department of Computer Science and Engineering: two faculty members and one CSE student. All had academic familiarity with Bangla grammar and NLP-related annotation tasks. Before annotation, they followed the written guideline described above, which included category definitions, diagnostic cues, representative examples, and majority-voting rules. Annotation was performed independently to reduce individual bias. Each annotator first read the full sentence, identified the dominant finite verb, examined the surface subject and other major participants, determined whether the surface subject functioned as an agent, patient/theme, or affected entity, checked for passive markers or “”-marked agents, and then assigned one label: Active, Passive, or Middle. Ambiguous sentences were marked for review, and disagreements were resolved through majority voting during validation. Representative annotator-level examples showing individual labels, final majority-vote decisions, agreement types, and confidence scores are provided in Table 5.

**Table 5:** Representative annotator-level examples showing majority voting and confidence scoring.

The final label was determined using a majority-voting protocol [13] among three independent annotators. Full agreement occurred when all three annotators assigned the same label, producing a confidence score of 1.00. Majority agreement occurred when at least two annotators assigned the same label, producing a confidence score of 0.67. This 2-of-3 agreement criterion was used as the operational acceptance threshold for final label assignment. The confidence score was computed as:

(1) $$Confidence\;Score=\frac{Number\;of\;Annotators\;Agreeing}{Total\;Number\;of\;Annotators}$$

Here, a higher Confidence score indicates stronger consensus. Ambiguous or structurally complex sentences were reviewed by a senior linguistic reviewer before final inclusion in the dataset. To quantify annotation reliability, Inter-Annotator Agreement (IAA) was calculated using Fleiss’ Kappa (κ), defined as:

(2) $$\kappa =\frac{\bar{P}-\bar{P_{e}}}{1-\bar{P_{e}}}$$

Here $\bar{P}$ represents the observed agreement among annotators, and $\bar{P_{e}}$​ represents the probability of agreement expected by chance. A higher κ value indicates stronger agreement among annotators and supports the robustness of the labelling process. Fleiss’ Kappa was computed across the complete set of 4397 annotated sentence instances, not on a sampled subset. The calculation used the independent labels assigned by all three annotators for each sentence. The resulting overall Fleiss’ Kappa value was κ = 0.955, indicating very high inter-annotator reliability and exceeding the substantial-agreement cut-off of κ ≥ 0.61. In the final annotation file, 4199 sentences reached full agreement and 198 sentences reached majority agreement. As shown in Table 6, among the 198 majority-resolution cases, disagreements occurred mainly between Active and Passive constructions (79 cases, 39.90%), followed by Passive and Middle constructions (63 cases, 31.82%) and Active and Middle constructions (56 cases, 28.28%). No instance showed three-way disagreement among Active, Passive, and Middle labels. This indicates that annotator uncertainty was distributed across multiple class boundaries, with a notable proportion involving Passive and Middle constructions, where surface cues and event-oriented interpretations may overlap.

**Table 6:** Class-pair distribution of majority-resolution annotation disagreements.

Disagreement pair	Number of cases	Percentage of majority-resolution cases
Active vs Passive	79	39.90%
Passive vs Middle	63	31.82%
Active vs Middle	56	28.28%
Active vs Passive vs Middle	0	0.00%
Total	198	100%

### 4.4 Data validation

**Fig. 9:**
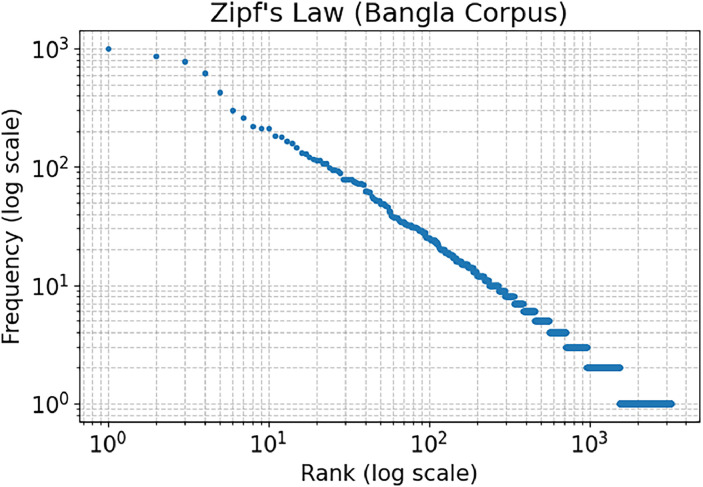
Zipf’s law in the Bangla voice corpus: log–log rank–frequency distribution of tokens.

To evaluate the lexical authenticity and statistical characteristics of the Bangla voice corpus, we first examined its rank–frequency distribution using Zipf’s law. The corpus comprises 18,794 total tokens and 3234 unique tokens, indicating substantial lexical variability. The log–log rank–frequency distribution (Fig. 9) exhibits a clear power-law behaviour consistent with natural language corpora. A small number of high-frequency tokens dominate the corpus, including “” (1000 occurrences in the full corpus, including 978 occurrences in Passive sentences), “” (874), and “” (779), forming the steep head of the distribution. As rank increases, the distribution transitions into an approximately linear region in log–log space, followed by a long tail composed of low-frequency tokens. Notably, approximately 52.4% of unique tokens occur only once (hapax legomena), reflecting high lexical diversity. Linear regression performed on log(rank) versus log(frequency) yielded a Zipf exponent of s ≈ 0.98–0.99 with a goodness-of-fit of R² ≈ 0.99, confirming strong adherence to Zipfian behaviour. These findings validate that the corpus preserves the statistical structure characteristic of naturally occurring Bangla language data.

Beyond lexical validation, we further assessed the discriminative capability of the dataset through supervised classification experiments. Six widely used linear and probabilistic classifiers such as Logistic Regression, LinearSVC, SGD (log_loss), Ridge, Multinomial Naïve Bayes, and Complement Naïve Bayes, were evaluated on the three-class voice categorization task (Active, Passive, Middle). For supervised benchmarking, the dataset was divided into stratified training and test sets using an 80:20 split with a random seed of 42. Bangla sentences were represented using character-level TF-IDF features with 3–5-character n-grams and a minimum document frequency of 2. Model performance was measured using accuracy, precision, recall, and F1-score. As shown in Table 7, LinearSVC achieved the highest overall performance (accuracy = 0.9318, F1-score = 0.9310), followed closely by Ridge (accuracy = 0.9284). Logistic Regression and SGD (log_loss) also demonstrated competitive results, whereas MultinomialNB and ComplementNB produced comparatively lower performance (accuracy < 0.87). These results indicate that margin-based linear classifiers are particularly well suited to the feature representation of the dataset.

**Table 7:** Performance of supervised machine learning models on the Bangla voice classification task.

Model	Accuracy	Precision	Recall	F1-Score
Logistic Regression	0.9148	0.9141	0.9139	0.9130
LinearSVC	**0.9318**	**0.9311**	**0.9314**	**0.9310**
SGD (log_loss)	0.9170	0.9164	0.9163	0.9155
Ridge	0.9284	0.9276	0.9278	0.9275
MultinomialNB	0.8636	0.8772	0.8599	0.8591
ComplementNB	0.8670	0.8793	0.8632	0.8613

**Fig. 10:**
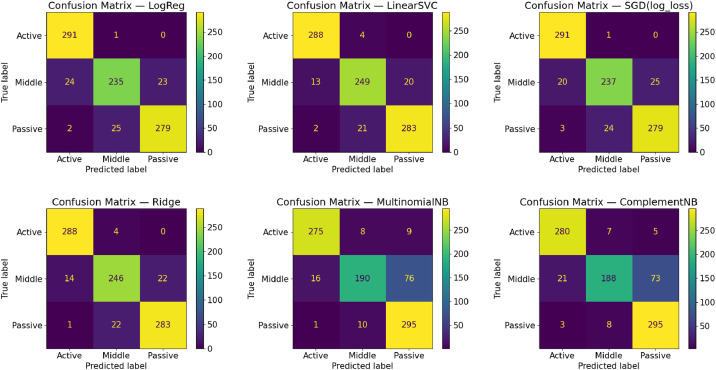
Comparative confusion matrices illustrating class-wise prediction performance across machine learning models.

Class-wise prediction behaviour is illustrated in the confusion matrices (Fig. 10). Strong diagonal dominance is observed for LinearSVC and Ridge, indicating consistent and balanced classification across the three voice categories. Minor misclassifications primarily occur between the Middle and Passive classes, while Naïve Bayes models exhibit relatively higher off-diagonal values.

**Fig. 11:**
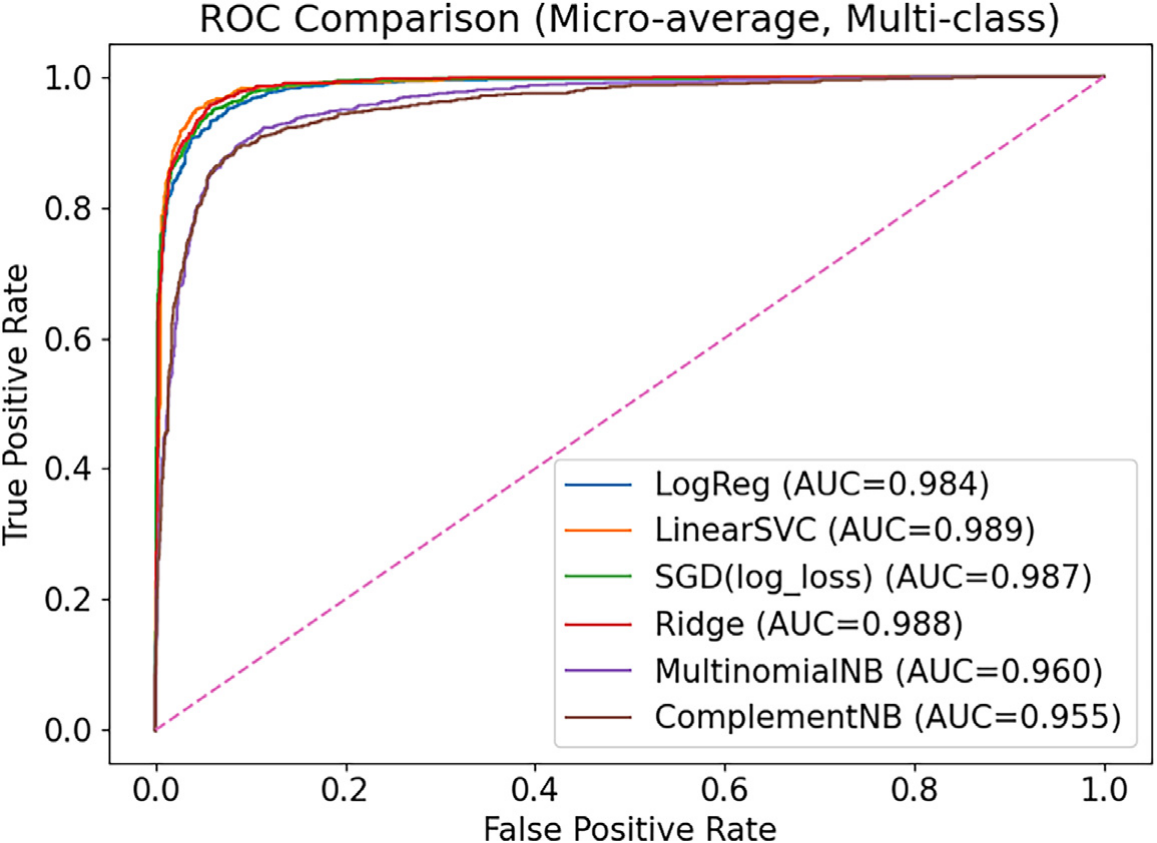
Comparative micro-averaged ROC analysis of supervised classifiers on the Bangla voice dataset.

Finally, micro-averaged ROC analysis (Fig. 11) further confirms the dataset’s separability. All classifiers achieved AUC values above 0.95, with LinearSVC attaining the highest AUC (0.989), followed by Ridge (0.988) and SGD (0.987). The consistently high AUC values demonstrate strong multi-class discriminative capability and reinforce the robustness of the proposed Bangla voice dataset for supervised machine learning applications.

The Zipfian distribution and consistently high classification performance confirm that the Bangla voice dataset maintains natural linguistic structure and supports reliable supervised learning, validating its suitability for computational analysis and voice classification research.

## Limitations

The BanglaVoice dataset is a curated, sentence-level resource and therefore does not aim to represent the full syntactic and lexical variability of Bangla. Sentences were restricted to structurally complete clauses containing a single dominant finite verb to ensure unambiguous voice annotation; as a result, complex multi-clausal and embedded constructions are not included. The corpus was compiled from contemporary written digital sources (2023–2025), and spoken language, strong dialectal variation, and historical texts are not explicitly represented. Although annotation was conducted through a multi-annotator protocol with strong agreement, Middle constructions may involve subtle interpretive distinctions inherent to linguistic analysis. This is also reflected in the majority-resolution analysis, where 63 cases, or 31.82%, involved Passive–Middle disagreement, suggesting that these non-active constructions may share surface-level auxiliary or event-oriented cues in some contexts. Additionally, the dataset provides voice labels only and does not include deeper syntactic layers such as dependency or semantic role annotations.

A further limitation is that some voice categories, particularly Passive constructions, may contain frequent surface cues such as “” and auxiliary forms. Although these cues were used only as annotation guidance, trained classifiers may learn lexical-marker associations rather than deeper grammatical voice generalizations. Consequently, model performance may decrease on Passive sentences that do not contain overt passive markers. Future work should include more cue-diverse Passive and Middle constructions and evaluate models on cue-controlled test subsets. Despite these constraints, the dataset offers a balanced and statistically validated foundation for sentence-level grammatical voice research in Bangla NLP.

## Ethics Statement

The authors confirm that this work complies with the ethical requirements for publication in Data in Brief. The BanglaVoice dataset was constructed exclusively from publicly accessible Bangla digital text sources, including news portals, magazines, blogs, and literary platforms. No human participants were directly involved in the study, and no private, confidential, or personally identifiable information was collected or processed. The dataset contains only publicly available written text used for linguistic analysis and computational research purposes. Therefore, formal ethical approval and informed consent were not required for this study.

## CRediT Author Statement

**Md. Jahidul Alam**: Conceptualization, Methodology, Project administration, Supervision, Data curation, Writing – review & editing. **Zannatul Mawa Koli**: Conceptualization, Methodology, Formal analysis, Software, Data curation, Visualization, Writing – original draft, Writing – review & editing. **Labony Sur**: Investigation, Validation, Data curation, Writing – review & editing. **Zahara Al Zarin**: Investigation, Validation, Data curation, Writing – review & editing. **Most. Hasna Hena**: Investigation, Validation, Data curation. **Tapasy Rabeya**: Investigation, Validation, Data curation.

## Data Availability

Mendeley DataBanglaVoice(Active, Passive, Middle) (Original data)
